# Hydrophilic Nonwoven Nanofiber Membranes as Nanostructured Supports for Enzyme Immobilization

**DOI:** 10.1021/acsapm.2c00863

**Published:** 2022-07-22

**Authors:** Antonio L. Medina-Castillo, Lucija Ruzic, Bernd Nidetzky, Juan M. Bolivar

**Affiliations:** † Nanomateriales y Polimeros S.L. (NanoMyP®), Spin-Off Company of the University of Granada, BIC Building, Avd. Innovacion 1, E-18016 Granada, Spain; ‡ Department of Analytical Chemistry, University of Granada, Avd. Fuentenueva s/n, 18071 Granada, Spain; § FQPIMA Group, Chemical and Materials Engineering Department, Faculty of Chemical Sciences, Complutense University of Madrid, 28040 Madrid, Spain; ∥ Institute of Biotechnology and Biochemical Engineering, Graz University of Technology, NAWI Graz, Petersgasse 12, A-8010 Graz, Austria; ⊥ Austrian Centre of Industrial Biotechnology, Krenngasse 37, A-8010 Graz, Austria

**Keywords:** copolymerization, controlled/living radical polymerization, nonwoven nanofiber membranes, enzyme immobilization, biocatalyst

## Abstract

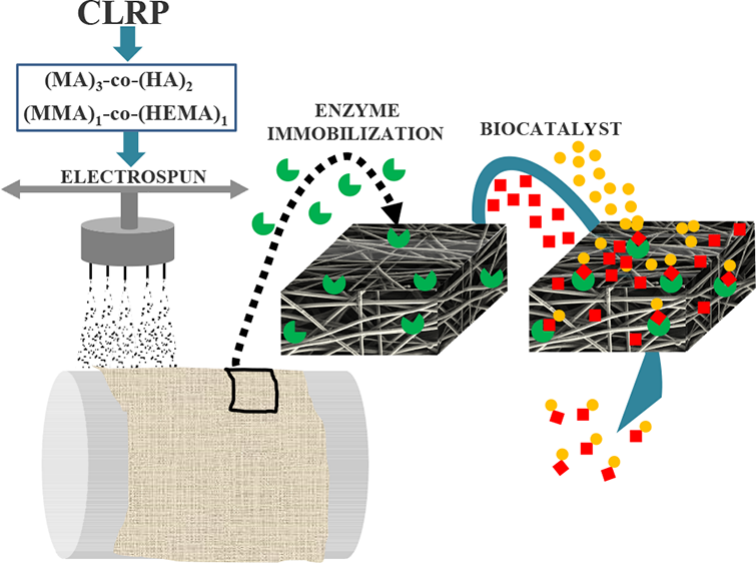

The high porosity, interconnected pore structure, and high surface area-to-volume ratio make the hydrophilic nonwoven nanofiber membranes (NV-NF-Ms) promising nanostructured supports for enzyme immobilization in different biotechnological applications. In this work, NV-NF-Ms with excellent mechanical and chemical properties were designed and fabricated by electrospinning in one step without using additives or complicated crosslinking processes after electrospinning. To do so, two types of ultrahigh-molecular-weight linear copolymers with very different mechanical properties were used. Methyl methacrylate-*co*-hydroxyethyl methacrylate (p(MMA)-*co*-p(HEMA)) and methyl acrylate-*co*-hydroxyethyl acrylate (p(MA)-*co*-p(HEA)) were designed and synthesized by reverse atom transfer radical polymerization (reverse-ATRP) and copper-mediated living radical polymerization (Cu^0^-MC-LRP), respectively. The copolymers were characterized by nuclear magnetic resonance (^1^H-NMR) spectroscopy and by triple detection gel permeation chromatography (GPC). The polarity, topology, and molecular weight of the copolymers were perfectly adjusted. The polymeric blend formed by (MMA)_1002_-*co*-(HEMA)_1002_ (*M*_w_ = 230,855 ± 7418 Da; *M*_n_ = 115,748 ± 35,567 Da; PDI = 2.00) and (MA)_11709_-*co*-(HEA)_7806_ (*M*_w_ = 1.972 × 10^6^ ± 33,729 Da; *M*_n_ = 1.395 × 10^6^ ± 35,019 Da; PDI = 1.41) was used to manufacture (without additives or chemical crosslinking processes) hydroxylated nonwoven nanofiber membranes (NV-NF-Ms-OH; 300 nm in fiber diameter) with excellent mechanical and chemical properties. The morphology of NV-NF-Ms-OH was studied by scanning electron microscopy (SEM). The suitability for enzyme binding was proven by designing a palette of different surface functionalization to enable both reversible and irreversible enzyme immobilization. NV-NF-Ms-OH were successfully functionalized with vinyl sulfone (281 ± 20 μmol/g), carboxyl (560 ± 50 μmol/g), and amine groups (281 ± 20 μmol/g) and applied for the immobilization of two enzymes of biotechnological interest. Galactose oxidase was immobilized on vinyl sulfone-activated materials and carboxyl-activated materials, while laccase was immobilized onto amine-activated materials. These preliminary results are a promising basis for the application of nonwoven membranes in enzyme technology.

## 1 Introduction

Electrospinning is a relatively simple and versatile technique for preparing continuous fibers with diameters ranging from tens of nanometers to several micrometers.^1,2^ The resulting membranes feature interconnected pores and usually possess higher porosities, higher surface roughness, and larger effective surface areas than conventional polymeric and ceramic membranes.^3,4^ These advantages make electrospun membranes very useful in filtration processes and tissue engineering and as carrier support in the preparation of immobilized enzymes.^5−8^

Immobilized enzymes show application in different sectors of biotechnology, biosensing, and catalysis in chemical production.^5−8^ The enzyme immobilization approach involves the incorporation of the enzyme into a prefabricated solid material, the *in situ* enzyme encapsulation/entrapment, or the immobilization into *ex novo* solid supports (CLEAs, nanoflowers, etc.).^9−11^ The primary function of enzyme immobilization is simplifying enzyme handling and enabling the reuse or continuous use of the enzyme. Besides these technical advantages, enzyme immobilization is usually associated with the possibility of modulating enzyme properties such as activity, stability, selectivity, etc.; in that way, the science of enzyme immobilization can provide a powerful toolbox to suit enzyme biocatalysts to application requirements. The design of an enzyme-immobilized biocatalyst with practical use and suitable functional properties involves a multiparameter process.^9−11^

One key aspect of the enzyme immobilization preparation is the selection of material and the physicochemical principles of enzyme incorporation, which dictates the suitability of the use and the properties of the biotechnological asset.^9−12^ The material features have a critical influence on the choice of the reactor, conditions of application, and also functional properties of the immobilized enzyme such as activity and stability.^9−13^ It is therefore not surprising that the enzyme technology field has been adopting trends in material engineering,^14−17^ where the new generation of materials has brought advances regarding new structural features and functionalities, carrying the applicability of enzymes beyond traditional formats of use.^18−20^ In this sense, inorganic and organic polymeric membranes have been receiving considerable attention.^21−23^

Among new materials, nonwoven nanofiber membranes (NV-NF-Ms) have shown to be one of the most desirable nanostructured supports for enzyme immobilization due to their physicochemical properties.^23−26^ Surface features (hydrophobic/hydrophilic properties) and surface functionalization to control enzyme binding are important design aspects to achieve practical, active, and stable catalysts. The suitable balance of hydrophobic/hydrophilic character should be normally suited to specific requirements of enzyme and reaction features. While hydrophobic membranes are easily obtained and available, hydrophilic NV-NF-Ms are normally hindered by poor mechanical properties such as abrasion resistance, tensile strength, elongation at break, and temperature resistance, among others, attributed to their high porosity, intrinsically low, random fiber orientations, and weak interactions between fiber junctions.^3,4,27−30^

To achieve hydrophilic NV-NF-Ms with suitable mechanical properties, different design alternatives are possible. Although the polarity and topology of electrospinnable polymers are normally considered parameters that affect the mechanical properties of NV-NF-Ms, the molecular weight (*M*_w_) is indeed one of the most important factors in the tensile strength and elongation at break of NV-NF-Ms. The positive relationship between *M*_w_ and mechanical properties of NV-NF-Ms can be ascribed to an increase in the length of the polymer chains.^31−34^ However, the difficulty of synthesizing well-controlled electrospinnable hydrophilic polymers means that, in most cases, hydrophilic NV-NF-Ms are hindered by poor mechanical strength.^35,36^ Today, most of the efforts aimed at improving the mechanical properties of hydrophilic NV-NF-Ms are focused on the use of additives during the electrospinning process and chemical crosslinking processes after electrospinning.^3,4,27−30^ Less effort is devoted to designing electrospinnable polymers capable of producing hydrophilic NV-NF-Ms with enhanced mechanical properties without the need for additives or chemical crosslinking processes.^37−40^

In this work, methacrylate and acrylate electrospinnable, ultrahigh-molecular-weight copolymers have been designed and synthesized by reverse-ATRP and Cu^0^-MC-LRP,^40−46^ respectively. Reverse-ATRP is a kind of copper-mediated living radical polymerization. By and large, the main differences between reverse-ATRP and Cu^0^-MC-LRP are that, in reverse-ATPR, a conventional radical initiator, usually a thermal initiator such as 2,2′-azobis(2-methylpropionitrile), is used to start the polymerization and, in Cu^0^-MC-LRP, a radical initiator is not used; further, a catalytic amount of Cu^0^ is used as a reducing agent to keep the concentration of Cu^+^ constant throughout the polymerization. Methacrylate and acrylate copolymers are chemically similar (totally miscible to each other in polar solvents) but with very different mechanical properties. Methacrylate copolymers are very hard with null elasticity, and acrylate copolymers are soft and gummy with high elasticity. All the copolymers were characterized by ^1^H-NMR and by triple detection gel permeation chromatography (GPC). The polarity, topology, and molecular weight of the copolymers have been carefully adjusted to maintain their complete insolubility in aqueous media and high solubility in the most common polar organic solvents used in electrospinning (dimethyl sulfoxide, dimethylformamide, *N*-methyl pyrrolidone, etc.). Then, we show that the polymeric blend (MMA)_1002_-*co*-(HEMA)_1002_/(MA)_11709_-*co*-(HEA)_7806_ (50/50, w/w) is an ideal candidate for the fabrication by electrospinning (without additives or chemical crosslinking processes) of hydroxylated nonwoven nanofiber membranes NV-NF-Ms-OH with excellent mechanical and chemical properties.

Besides mechanical properties, the feasibility to generate different surface binding functional groups is key for the practical application of material immobilization supports. To validate the usefulness of the membranes for enzyme immobilization, NV-NF-Ms-OH were functionalized with vinyl sulfone, amine, and carboxyl groups. The palette of selected functional groups covers both irreversible (covalent) and ion exchange (reversible) immobilization.^11,47,48^ For covalent irreversible immobilization, a procedure of functionalization of NV-NF-Ms-OH with vinyl sulfone groups (RSOOCH_2_CH_2_) was established. The reaction of divinyl sulfone with the primary hydroxyl groups localized on NV-NF-Ms-OH allows the introduction of vinyl sulfone function on the surface of the fibers. Then, RNH_2_ or RSH groups of enzymes can react in mild conditions (pH = 7; compatible with the biological nature of the enzymes) with the vinyl sulfone groups, providing covalent coupling of enzymes by a Michael-type reaction.^46^ Vinyl sulfone is a powerful versatile binding group that is able to quickly react with multiple nucleophilic residues from the protein surface.^49−53^ Amine and carboxyl groups are driving groups for ionic adsorption based on anion and cation exchange, respectively.^11,47,48^ Amine groups also enable the generation of heterofunctional supports by previous modification with glutaraldehyde, which is useful in achieving covalent immobilization.^54^ Finally, post-immobilization treatments, e.g., covering with ionic polymers, are a useful strategy for further stabilization of immobilized enzymes previously immobilized.^55,56^ This toolbox was applied for the immobilization of two enzymes of biotechnological interest as galactose oxidase and laccase.^57−64^ Laccases have attracted proven interest in biosensing and bioremediation, and they receive increasing attention in biocatalytic synthesis.^57,59,60,62−64^ Galactose oxidase finds application in biosensing, production of carbohydrate-based reactive aldehydes, and valorization of hydroxymethylfurfural.^58,64^ These preliminary results are a promising basis for the application of nonwoven membranes in enzyme technology.

## 2 Materials and Methods

### 2.1 Chemicals

Methyl acrylate (MA), 2-hydroxyethyl acrylate (HEA), methyl methacrylate (MMA), 2-hydroxyethyl methacrylate (HEMA), 2,2′-azobis(2**-**methylpropionitrile) (AIBN), methyl 2-bromopropionate (MBP), *N*,*N*,*N*′,*N*′,*N*″-pentamethyldiethylenetriamine (PMDETA), tris(2-dimethylaminoethyl)amine (M_6_-TREN), CuBr_2_, Cu^0^, *m*-xylene (*m*-Xi), dimethyl sulfoxide (DMSO), dimethylformamide (DMF), divinyl sulfone (DVS), ethylene diamine (EDA), dansyl cadaverine (DC), glutaraldehyde solution (50 wt % in H_2_O), 10% poly(ethyleneimine) solution (PEI; average *M*_n_ ∼ 60,000 by GPC, average *M*_w_ ∼ 750,000 by LS), potassium phosphate, sodium carbonate, 1% PEI (average *M*_w_ ∼ 25,000 by LS, average *M*_n_ ∼ 10,000 by GPC), glycine (GLY), galactose, 2,2′-azino-bis(3-ethylbenzothiazoline-6-sulfonic acid) (ABTS), galactose oxidase from *Dactylium dendroides*, laccase from *Trametes versicolor*, and horseradish peroxidase (HRP) were purchased from Sigma-Aldrich.

### 2.2 Synthesis of Well-Controlled Copolymers by Control Living Radical Polymerization (CLRP) Techniques

#### 2.2.1 Synthesis of Acrylate Copolymers by Cu^0^-MC-LRP

Acrylate copolymers p(MA)-*co*-p(HEA) with statistical topology, controlled polarity (concentration of hydroxyl groups), and ultrahigh molecular weight were synthesized by Cu^0^-MC-LRP. The Cu^0^-MC-LRP catalytic system used was as follows: methyl 2-bromopropionate as an initiator, tris(2-dimethylaminoethyl) amine as a ligand, copper/copper(II) as transition metals (MBP/M_6_-TREN/Cu^0^/CuBr_2_), and dimethyl sulfoxide (DMSO) as a solvent.

Cu^0^-MC-LRP is very sensitive to any trace of impurities. On the other hand, the traces of ethylene glycol diacrylate (EDMA; crosslinker) formed by the condensation of the HEA molecules during storage must also be eliminated prior to polymerization to avoid the crosslinking. Thus, the monomers HEA and MA were previously purified.

##### 2.2.1.1 HEA Purification Protocol

HEA was passed through a basic alumina column to eliminate the inhibitor. To remove traces of the hydrophobic EDMA, 70 mL of HEA (previously passed through a basic alumina column) was dissolved in 210 mL of distilled water, and EDMA was extracted by 11 liquid–liquid extractions with 210 mL of hexane. Subsequently, 58 g of NaCl was dissolved in the monomer aqueous solution, and HEA was extracted by 5 liquid–liquid extractions with 200 mL of diethyl ether. Then, traces of water in the diethyl ether solution were removed by adding 300 g of anhydrous sodium sulfate; the solutions were stirred for a few minutes and filtered. Diethyl ether was completely evaporated in a rotavapor, and the purified HEA was stored at −20 °C.

##### 2.2.1.2 MA Purification Protocol

The monomer MA was passed through a column of basic alumina to eliminate the inhibitor and stored at −20 °C.

As shown in Table S1, six copolymers with different feed molar ratios (%HEA/%MA), 10/90, 15/85, 25/75, 34/66, 45/55, and 55/45, were synthesized by Cu^0^-MC-LRP.

##### 2.2.1.3 Cu^0^-MC-LRP Protocol

The total mass of the monomers remained constant in all cases (MA + HEA = 59.2700 g). Monomers were added into 50 mL Schlenk flasks, and then in the following order, 59.2700 g of DMSO, 0.0020 g of Cu^0^, 0.0160 g of tris[2-(dimethylamino)ethyl]amine (M_6_-TREN), 0.0012 g of CuBr_2_, and 0.0060 g of methyl 2-bromopropionate (MBP) were added into the flasks. The flasks were closed with a septum, the oxygen was removed by bubbling nitrogen for a few minutes, and then four freeze–pump–thaw cycles were carried out (after the last freeze–pump–thaw cycle, the flasks were filled with nitrogen). Subsequently, the sealed flasks were placed in a thermostatic oil bath at 25 °C for 24 h with stirring. Then, the copolymers were purified by dissolving in acetone and precipitating them in distilled water (two times). After purification, the copolymers were dried in a vacuum at 80 °C to a constant weight.

#### 2.2.2 Synthesis of Methacrylate Copolymers by Reverse-ATRP

The reverse-ATRP catalytic system used was as follows: 2,2′-azobis(2-methylpropionitrile) (AIBN) as an initiator, *N*,*N*,*N*′,*N″*,*N*″-pentamethyldiethylenetriamine (PMDETA) as a ligand, copper(II) as a transition metal, and a mixture of dimethyl sulfoxide (DMSO)/*m*-Xi as a solvent. The monomers HEMA and MMA were purified following the same protocols as those used for HEA and MA.

##### 2.2.2.1 Reverse-ATRP Protocol

In a 500 mL two-necked flask equipped with reflux and a magnetic stirrer were added 95.00 mL of DMSO, 0.14 g of CuBr_2_, 0.24 g of *N*,*N*,*N*′,*N″*,*N″*-pentamethyldiethylenetriamine **(**PMDETA), 90.08 g of MMA, 40.12 g of HEMA, and 0.23 g of 2,2′-azobis(2**-**methylpropionitrile) (AIBN) dissolved in 60.02 g of xylene. The mixture was stirred at 250 rpm; when all the components were completely dissolved, the reaction mixture was cooled at 0 °C and purged with highly pure nitrogen for 20 min. Then, the reaction was carried out at 80 °C in an oil bath for 6 h. After polymerization, the copolymers were purified by dissolving acetone and precipitating them in distilled water three times. Then, they were dried in a vacuum at 80 °C to a constant weight.

### 2.3 Electrospinning of Highly Hydroxylated Nonwoven Membranes NV-NF-Ms-OH

The w/w ratios of polymeric blend (MMA)_1002_-*co*-(HEMA)_1002_/(MA)_11709_-*co*-(HA)_7806_ selected to be processed by electrospinning were 0/100, 25/75, 50/50, 75/25, and 100/0. Copolymers were dissolved in DMF, loaded into 20 cm^3^ Teflon syringes (Becton & Dickinson), and extruded through a 10-needle (stainless-steel capillary tube with outer and inner diameters of 1.5 and 1.1 mm, respectively) head coupled to a mechanical axis with axial movement. The flow rates and voltages were selected to allow dry fibers in nonwoven mats, and the fibers were collected on a rotary drum collector. Figure S1 shows the setup and the electrospinning processing parameters.

### 2.4 Functionalization of NV-NF-Ms-OH

The hydroxyl and ester groups of NV-NF-Ms-OH were used for their subsequent functionalization.

#### 2.4.1 Functionalization of NV-NF-Ms-OH with Vinyl Sulfone Groups, NV-NF-Ms-VS

A piece of the membrane (16 × 11cm) was introduced into 70 mL of a solution of DVS (0.33 M) in sodium carbonate buffer (333 mM) at pH = 12.50 for 2 h. Subsequently, the membranes were washed three times with distilled water for 15 min and dried at 50 °C in a vacuum oven.

#### 2.4.2 Functionalization of NV-NF-Ms-OH with Carboxyl Groups, NV-NF-Ms-COOH

Basic hydrolysis of ester groups (R-COOCH_3_ and RCOOCH_2_CH_2_OH) present on the surface of the fibers was carried out by introducing a piece of the membrane (16 × 11 cm) into 70 mL of a solution of sodium carbonate (333 mM) at pH = 12.50 during 30 min. Then, the membranes were washed three times with distilled water and dried at 50 °C in a vacuum oven.

#### 2.4.3 Functionalization of NV-NF-Ms-VS with Amine Groups, NV-NF-Ms-NH_2_

Vinyl sulfone groups can react easily with amine groups in mild conditions by a Michael-type reaction. Thus, to functionalize the membranes with amine groups, a piece of NV-NF-Ms-VS (16 × 11 cm) was introduced into 70 mL of a solution of ethylene diamine (0.33 M) in phosphate buffer (100 mM) at pH = 8 for 4 h. Subsequently, the membranes were washed three times with distilled water and dried at 50 °C in a vacuum oven.

### 2.5 Measurement of Enzyme Activity

The activity was measured by monitoring the initial oxygen consumption rates. Oxygen concentration was quantified by using a robust oxygen micro optical oxygen meter FireStingO2. The final reaction volume was 5 mL. In the case of galactose oxidase, a reported procedure was followed.^64^ The reaction mixture consisted of 250 mM galactose, 0.02 mg/mL HRP, 0.00028 mg/mL galactose oxidase, and 25 mM potassium phosphate buffer (pH 7) at 25 °C. One unit (U) of enzymatic activity was determined as 1 μmol of oxygen consumed per minute. As reference for immobilization reporting, unit and mg pro protein were used. For enzyme preparation, the commercial enzyme powder was resuspended in 25 mM sodium phosphate at pH 7.0.

As an alternative approach, colorimetric assays were performed. For galactose oxidase, the immobilized enzyme was measured with an offline spectrophotometric method that includes coupled reaction with horseradish peroxidase (HRP) as well as the use of a mediator 2,2′-azino-bis(3-ethylbenzothiazoline-6-sulfonic acid) (ABTS). The activity was determined by measuring the increase in absorbance at 420 nm produced by the formation of ABTS radicals and characterized as the amount of enzyme necessary to produce 2 μmol of ABTS^+^ per min. The reaction was performed in a plastic Petri dish (85 mm in diameter). A total of 35 cm^2^ (approx. 30 mg) of the biocatalyst (enzyme immobilized on the nanomembrane) was immersed in the 40 mL reaction mixture and stirred on a roller mixer at 40 rpm. One milliliter of sample was taken every minute, and absorbance was measured at 420 nm. The reaction mixture consisted of 250 mM galactose, 0.02 mg/mL HRP, 1 mM ABTS, immobilized enzyme, and 25 mM potassium phosphate buffer (pH 7) at 25 °C. For laccase, the activities of the soluble and immobilized enzymes were determined by measuring the increase in absorbance at 420 nm and were characterized as the amount of enzyme required to oxidize 1 μmol of ABTS in 1 min. The reaction with the immobilized enzyme was performed as an offline method in a plastic Petri dish (85 mm in diameter). A total of 23 cm^2^ (approx. 20 mg) of the biocatalyst (enzyme immobilized on the nanomembrane) was immersed in the 40 mL reaction mixture and stirred on a roller mixer at 40 rpm. One milliliter of sample was taken every minute, and absorbance was measured at 420 nm. The reaction mixture consisted of 0.5 mM ABTS, 0.005 mg/mL laccase, and 25 mM potassium phosphate buffer (pH 6) at 25 °C. Commercial preparation of galactose oxidase displayed an activity of 0.54 U/mg powder and 1.5% of protein purity. As reference for immobilization reporting, unit and mg pro protein were used. Commercial preparation of laccase displayed an activity of 2.5 U/mg powder and 2.5% of protein purity.

### 2.6 Enzyme Immobilization

#### 2.6.1 Covalent Immobilization of Galactose Oxidase on NV-NF-Ms-NH_2_ Preactivated with Glutaraldehyde

For the covalent immobilization of the commercial preparation of galactose oxidase onto these membranes, it is necessary to preactivate them with glutaraldehyde. For that purpose, the membrane was divided into ∼30 mg pieces. Each piece was then activated with 10% glutaraldehyde solution (30 mg of the membrane immersed in 100 mL of glutaraldehyde solution, with gentle stirring for 18 h at 25 °C). After preactivation, the support was washed five times with 25 mM potassium phosphate buffer (pH 7.0), followed by one wash step with distilled water. Then, the membranes were immersed in the solution containing different amounts of galactose oxidase (7620, 1162, 94, and 21 U/g) dissolved in 25 mM sodium phosphate buffer (pH 7) and gently stirred for 3 h. After 3 h, the supernatant was separated, membranes were washed three times with buffer, and the activities of both the supernatant and membrane were tested.

#### 2.6.2 Electrostatic Immobilization of Laccase on NV-NF-Ms-NH_2_

Twenty milligrams of the membrane was immersed in 15 mL of 5 mM potassium phosphate at pH 6.0 containing 20 U/g of the enzyme. The mixture was gently stirred for 3 h at 25 °C. After the end of immobilization, the membrane was washed three times with buffer, and the activities of both the supernatant and membrane were tested.

#### 2.6.3 Electrostatic Immobilization of Galactose Oxidase on NV-NF-Ms-COOH

An amount of ∼30 mg of the membrane was immersed in 15 mL of 5 mM potassium phosphate buffer at pH 7.0 containing different amounts of enzyme (19–62 U/g). The mixture was gently stirred for 3 h at 25 °C. After the end of immobilization, membranes were washed three times with buffer and the activity in the supernatant was tested. For preventing enzyme leaking, the biocatalyst was treated with 10 and 1% poly(ethyleneimine) solution (PEI; average *M*_n_ ∼ 60,000 by GPC, average *M*_w_ ∼ 750,000 by LS, 50 wt % in H_2_O) and 1% PEI (average *M*_w_ ∼ 25,000 by LS, average *M*_n_ ∼ 10,000 by GPC). The mixture was kept under gentle stirring for ∼18 h at 25 °C, after which membranes were washed five times with 5 mM potassium phosphate buffer and five times with distilled water. The activity was tested*.*

#### 2.6.4 Covalent Immobilization of Galactose Oxidase on NV-NF-Ms-VS

An amount of ∼30 mg of the membrane was immersed in 15 mL of 25 mM potassium phosphate buffer at pH 7.0 containing different amounts of enzyme (350, 51, and 33 U/g). The mixture was gently stirred for 3 h at 25 °C. After the end of immobilization, membranes were washed three times with buffer and the activity in the supernatant was tested. Obtained biocatalysts were incubated in 10 mL of 100 mM sodium carbonate at pH 10 and 25 °C for 6 h to promote the enzyme-support multipoint covalent reaction. Biocatalysts were firmly washed with buffer and distilled water, after which the activity was tested.

## 3 Results and Discussion

### 3.1 Characterization of Acrylate and Methacrylate Copolymers: Formulation and Study of Their Blends

Unlike methacrylate polymers, which are extremely hard polymers that exhibit very low elongation at break,^65^ the acrylate copolymers usually behave as rubber with high flexibility and elongation at break. This can be explained because the absence of methyl groups in the main carbon chain of acrylates allows greater freedom of movement between chains; in the methacrylates, the methyl groups of the main carbon chain can behave as small branches that hinder the movement/fluidity between polymer chains. This small structural difference results in a large difference in their mechanical properties. In this work, we have exploited this structural difference to tune the mechanical properties of highly hydroxylated nonwoven nanofiber membranes NV-NF-Ms-OH. Keeping in mind that molecular weight (*M*_w_) is also a very important factor in the tensile strength and elongation at break of NV-NF-Ms-OH,^31−34^ acrylate and methacrylate copolymers with ultrahigh molecular weight and similar chemical composition were designed, synthesized, and characterized.

Cu^0^-MC-LRP has been extensively studied, and several propositions have been made about the mechanism in which Cu^0^ mediates controlled radical polymerization.^40,41^ This technique has proven to be a powerful tool for ultrafast polymerization of the hydrophobic monomers methyl acrylate, methyl methacrylate, and vinyl chloride, but it should be noted that, only in the case of methyl acrylate, ultrahigh molecular weights (greater than 500,000 Da) were achieved.^40,41^ To investigate the applications of Cu^0^-MC-LRP further, it was used in the copolymerization of the monomers methyl acrylate (MA) and 2-hydroxyethyl acrylate (HEA). First, a theoretical analysis of the copolymerization of MA and HEA was done by using the terminal model.^66^ The terminal model assumes that radical reactivity only depends on the terminal unit of the growing chain such that the molar fraction of monomer a in the copolymer (*F*_a_) depends only on monomer mole fractions (*f*_a_ and *f*_b_, with *f*_a_*+ f*_b_ = 1) and copolymerization reactivity ratios and is given by:
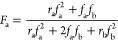
1where *r*_a_ and *r*_b_ are the copolymerization reactivity ratios of monomers a and b, respectively. The reactivity ratios used for MA and HEA were *r*_a_ = 0.94 and *r*_b_ = 0.90, respectively;^67^ since amphiphilic monomers like HEA exhibit different polymerization behavior in different solvents, inconsistencies exist between the different reported reactivity ratios for the same monomer pairs depending upon the reaction media and other conditions such as the preferential solvation of monomers around the active polymer radical.^68−70^ As summarized in Figure S2, the system runs through almost azeotropic copolymerization to any initial molar fraction of MA (*f*_0an_; *n* = 1, ..., 4), suggesting that copolymerization of MA and HAE provides polymeric chains with a homogeneous monomeric concentration.

The copolymers synthesized with the mixtures in Table S1 were characterized by triple detection gel permeation chromatography (GPC; Viscotek 270 Max of Malvern) and by ^1^H-NMR (Bruker Avance 400 MHz spectrometer). Figure S3 shows the GPC chromatographic profiles, and Table 1 shows the molecular weights (*M*_w_ and *M*_n_), yields, and polydispersities (PDIs). For the feed molar percentages of HEA of 45 and 55% (see Table S1), the yields were below 20%, and these copolymers were discarded. It is well known that spontaneous radical termination increases as the polarity of the monomers increases.^71,72^ Therefore, the high concentration of the polar monomer HEA in 45 and 55% cases could dramatically increase spontaneous radical terminations, reducing the lifetime of radical chains and thus the concurrent growth of all polymer chains. However, as shown in Table 1, for molar percentages of HEA between 10 and 34%, all the copolymers showed a dramatically high molecular weight and yields.

**Table 1:** Molecular Weights of Acrylate Copolymers p(MA)-*co*-p(HEA)

HEA feed molar %	*M*_w_ (Da)	*M*_n_ (Da)	yield (%)	*M̅*_w_ (Da)	*M̅*_n_ (Da)	PDI
10	1.162 × 10^6^	716,449	93	(11.56 ± 0.40847) × 10^5^	777,591 ± 53,003	1.49
	1.137 × 10^6^	805,809	95			
	1.168 × 10^6^	810,516	90			
15	6.039 × 10^6^	2.155 × 10^6^	89	(5.746 ± 0.653911) × 10^6^	(1.878 ± 0.395400) × 10^6^	3.08
	5.671 × 10^6^	1.721 × 10^6^	95			
	5.529 × 10^6^	1.759 × 10^6^	91			
25	3.167 × 10^6^	1.815 × 10^6^	98	(31.88 ± 0.88220) × 10^5^	(1.714 ± 0.220000) × 10^6^	1.86
	3.168 × 10^6^	1.714 × 10^6^	93			
	3.229 × 10^6^	1.614 × 10^6^	95			
34	1.980 × 10^6^	1.413 × 10^6^	90	(19.72 ± 0.33729) × 10^5^	(13.95 ± 0.35019) × 10^5^	1.41
	1.981 × 10^6^	1.418 × 10^6^	96			
	1.957 × 10^6^	1.355 × 10^6^	93			

Figure S4 shows the ^1^H-NMR spectra of the copolymers, and Table S2 summarizes the chemical composition of each copolymer calculated by the intensity ratio between signals **a** (CH_3_ of MA) and **b** (CH_2_–CH_2_ of HEA) of the ^1^H-NMR spectra. The high PDI and *M*_w_ in the case of the copolymer synthesized with an HEA feed molar percentage of 15% may be because, in this case, the removal of EDMA (crosslinker) during the purification of the HEA monomer (see Section 2.2.1) was not 100%: the presence of traces of EDMA during the polymerization can produce branching in the copolymers, giving place to the increase in both molecular weight and PDI.

As shown in Table S2, in all copolymers, the concentration of HEA is practically the same as the feed concentration, which agrees with the theoretical predictions of Figure S2. Acrylate copolymers in Table 1 presented water insolubility and a high-flexibility rubbery texture. On the other hand, they showed high solubility in dimethylformamide (DMF), dimethyl sulfoxide (DMSO), 1,4-dioxane, and NMP; above 6 wt %, the viscosity of the solutions was dramatically high due to their ultrahigh molecular weights. The lower viscosities were achieved in DMF, indicating that DMF is the best solvent for these copolymers (see Figure S5).

Methacrylate polymers with high molecular weights cannot be synthesized by Cu^0^-MC-LRP; the polymerization of methyl methacrylate by Cu^0^-MC-LRP results in molecular weights lower than 40,000 Da.^40,41^ We showed in a previous work^45^ that copolymerization of MMA and HEMA by reverse-ATRP leads to high-molecular-weight copolymers (150,000 Da). Thus, to synthesize a high-molecular-weight methacrylate copolymer miscible with (MA)_11709_-*co*-(HEA)_7806_ (with the same chemical composition; see Table S2), the copolymerization of MMA and HEMA was carried out by reverse-ATRP. The theoretical modeling of MMA and HEMA in Figure S6 suggests that this pair of monomers also provides polymeric chains with a homogeneous monomeric concentration.

Figure S7 shows the chromatographic profile and ^1^H-NMR spectrum of the methacrylic copolymer. The concentration of HEMA in the copolymer calculated by the intensity ratio between signals **a** (CH_3_ of MMA; 1.73) and **b** (CH_2_–CH_2_ of HEMA; 1.72) of ^1^H-NMR was 50%; in (MMA)_1002_-*co*-(HEMA)_1002_, the molecular weights by GPC were *M*_w_ = 230,855 ± 7418 Da and *M*_n_ = 115,748 ± 35,567 Da (PDI = 2.02), and the yield was 70%. The copolymer (MMA)_1002_-*co*-(HEMA)_1002_ was water-insoluble with a hard and brittle texture. The solubility of (MMA)_1002_-*co*-(HEMA)_1002_ was also tested in dimethylformamide (DMF), dimethyl sulfoxide (DMSO), 1,4-dioxane, and NMP, showing high solubility in all the solvents; above 38 wt %, the viscosity of the solutions was extremely high. Like in the case of acrylate copolymers, the lower viscosity was achieved in DMF, indicating that DMF is also the best solvent for (MMA)_1002_-*co*-(HEMA)_1002_.

To formulate electrospinnable polymeric blend (acrylate/methacrylate) solutions, the solubility between both copolymers (MMA)_1002_-*co*-(HEMA)_1002_ and (MA)_11709_-*co*-(HEA)_7806_ was studied in DMF. The (MMA)_1002_-*co*-(HEMA)_1002_/(MA)_11709_-*co*-(HEA)_7806_ w/w ratios tested were 10/90, 25/75, 50/50, 75/25, and 90/10, and the [(MMA)_1002_-*co*-(HEMA)_1002_ + (MA)_11709_-*co*-(HEA)_7806_]/solvent w/w ratio was 6/94. As shown in Figure S8, due to their similar chemical composition, both copolymers showed miscibility to each other in all ratios.

### 3.2 Electrospinning of the Polymeric Blend (MMA)_1002_-*co*-(HEMA)_1002_/(MA)_11709_-*co*-(HA)_7806_: Morphological Characterization and Physicochemical Properties of Nonwoven Nanofiber Membranes

**Figure 1:**
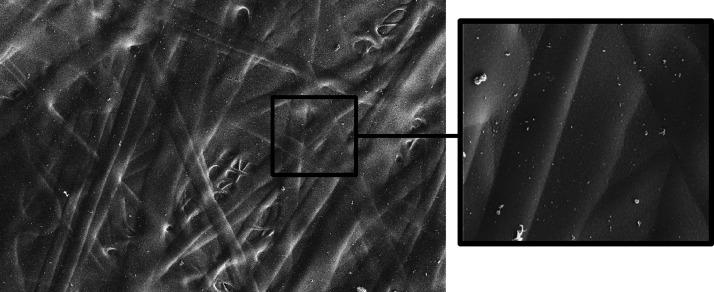
Electrospun copolymer (MA)_11709_-*co*-(HEA)_7806_.

Methacrylate ((MMA)_1002_-*co*-(HEMA)_1002_) and acrylate ((MA)_11709_-*co*-(HA)_7806_) copolymers were selected and used to manufacture highly hydroxylated nonwoven nanofiber membranes. The w/w ratios of selected blends (MMA)_1002_-*co*-(HEMA)_1002_/(MA)_11709_-*co*-(HA)_7806_ to be processed by electrospinning were 0/100, 25/75, 50/50, 75/25, and 100/0, and the blend/solvent (DMF) w/w ratio was 6/94. The morphological characterization was carried out by scanning electron microscopy (SEM). As shown in Figure 1, the copolymer (MA)_11709_-*co*-(HA)_7806_ provided elastic gummy membranes, in which the fibers are 100% fused together, forming a nonporous polymeric film.

**Figure 2:**
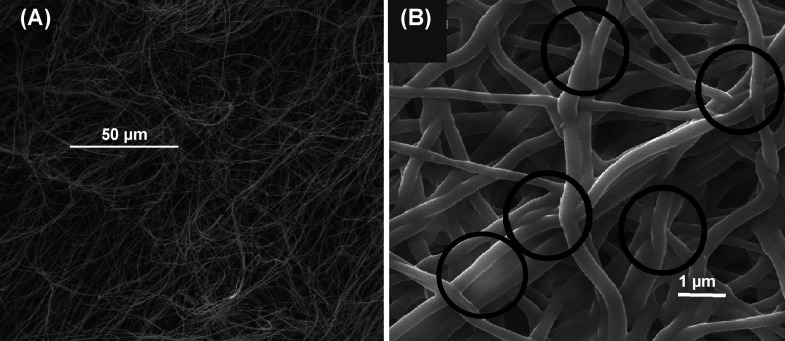
Electrospun copolymer (MMA)_1002_-*co*-(HMEA)_1002_ (A) and blend (MMA)_1002_-*co*-(HEMA)_1002_/(MA)_11709_-*co*-(HEA)_7806_ (25:75).

Figure 2 shows the membranes obtained with the copolymer (MMA)_1002_-*co*-(HEMA)_11,002_ (Figure 2A) and with the 25:75 blend (Figure 2B). The membrane manufactured with the copolymer (MMA)_1002_-*co*-(HEMA)_1002_ did not have any fusion point between fibers; they were completely loose, which provided membranes with a null abrasion resistance (hardly manipulatable materials, like a piece of nonprocessed cotton). The fusion between fibers in the membrane obtained with the blend (MMA)_1002_-*co*-(HEMA)_1002_/(MA)_11709_-*co*-(HEA)_7806_ (25:75) was no longer as high as in the case of the membrane manufactured with the copolymer (MA)_11709_-*co*-(HA)_7806_ (see Figure 1). But the fusion between fibers was still too high (see black circles in Figure 2B), providing very elastic membranes with a non-interconnected pore structure and thus with a low surface area-to-volume ratio.

**Figure 3:**
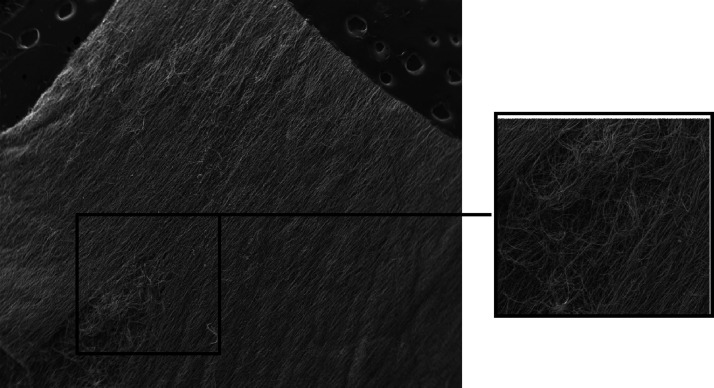
Electrospun blend (MMA)_1002_-*co*-(HEMA)_1002_/(MA)_11709_-*co*-(HEA)_7806_ (75:25).

As shown in Figure 3, the blend (MMA)_1002_-*co*-(HEMA)_1002_/(MA)_11709_-*co*-(HA)_7806_ (75:25) provided a compact nonwoven membrane with better abrasion resistance than the membrane obtained with the copolymer (MMA)_1002_-*co*-(HEMA)_1002_ (see Figure 2A), but the membrane was still very brittle and showed low abrasion resistance*.*

**Figure 4:**
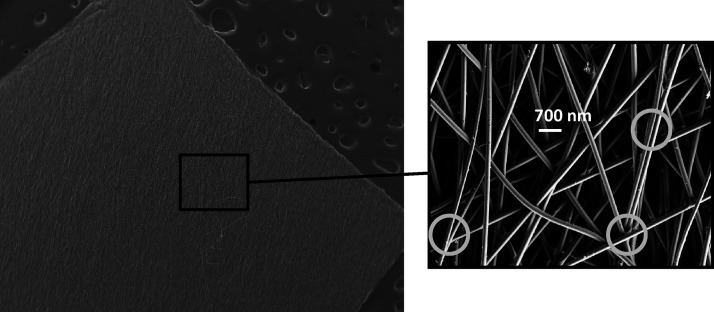
Electrospun blend (MMA)_1002_-*co*-(HEMA)_1002_/(MA)_11709_-*co*-(HEA)_7806_ (50/50).

The blend (MMA)_1002_-*co*-(HEMA)_1002_/(MA)_11709_-*co*-(HA)_7806_ (50/50, w/w) provided an optimal concentration of junction points between fibers and thus a compact membrane with an interconnected pore structure (see Figure 4) and excellent mechanical properties such as high abrasion resistance, high flexibility and elasticity, and high elongation at break, and it is easily manipulated: it can be cut, bent, twisted, etc.

Figure S9 shows a qualitative comparison of the elongation at break and abrasion resistance of membranes processed with blends (MMA)_1002_-*co*-(HEMA)_1002_/(MA)_11709_-*co*-(HA)_7806_ (75/25, w/w) (Figure S9B,D) and (MMA)_1002_-*co*-(HEMA)_1002_/(MA)_11709_-*co*-(HA)_7806_ (50/50, w/w) (Figure S9A,C).

**Figure 5:**
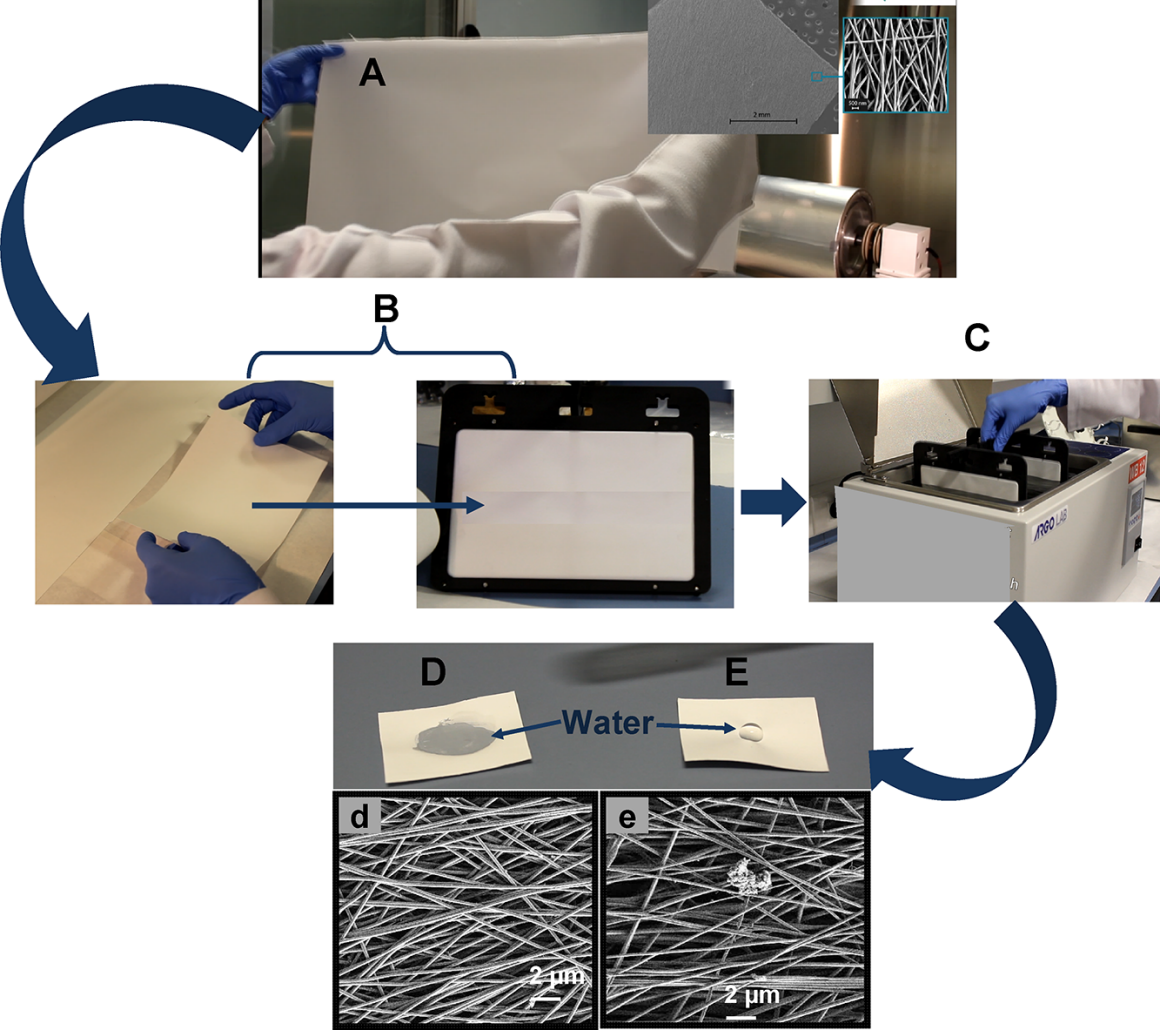
Membrane (30 × 60 cm) after 4 h of electrospinning (A), pieces of 11 × 16 cm fixed on frames (B), TT at 40 °C for 5 h (C), high degree of wetting after TT (*Q* = 2.06) (D), large contact angle before TT (*Q* = 0.00) (E), and morphology of the fibers after (d) and before (e) TT.

Just after electrospinning, the hydroxylated membrane fabricated with the blend (MMA)_1002_-*co*-(HEMA)_1002_/(MA)_11709_-*co*-(HA)_7806_ (50/50, w/w) was hydrophobic (see Figure 5E). It is well known that copolymers formed by hydrophilic and hydrophobic monomeric units can organize in hydrophobic and hydrophilic domains in the presence of water due to hydrophobic interactions.^73−75^ Thus, to reorient the hydrophilic domains and introduce amphiphilic properties in the membrane, a thermal treatment (TT) was carried out. After electrospinning, the membrane (30 × 60 cm; Figure 5A) was cut in pieces of 11 × 16 cm and they were fixed on frames to avoid wrinkles and deformations during TT (Figure 5B). Then, they were placed in a thermostatized bath at 40 °C for 5 h (Figure 5C). Subsequently, the membranes were dried in a vacuum oven at 50 °C; during TT, the reorientation of hydrophilic domains toward water molecules is produced, inducing amphiphilic properties in the membrane (see Figure 5D).

**Figure 6:**
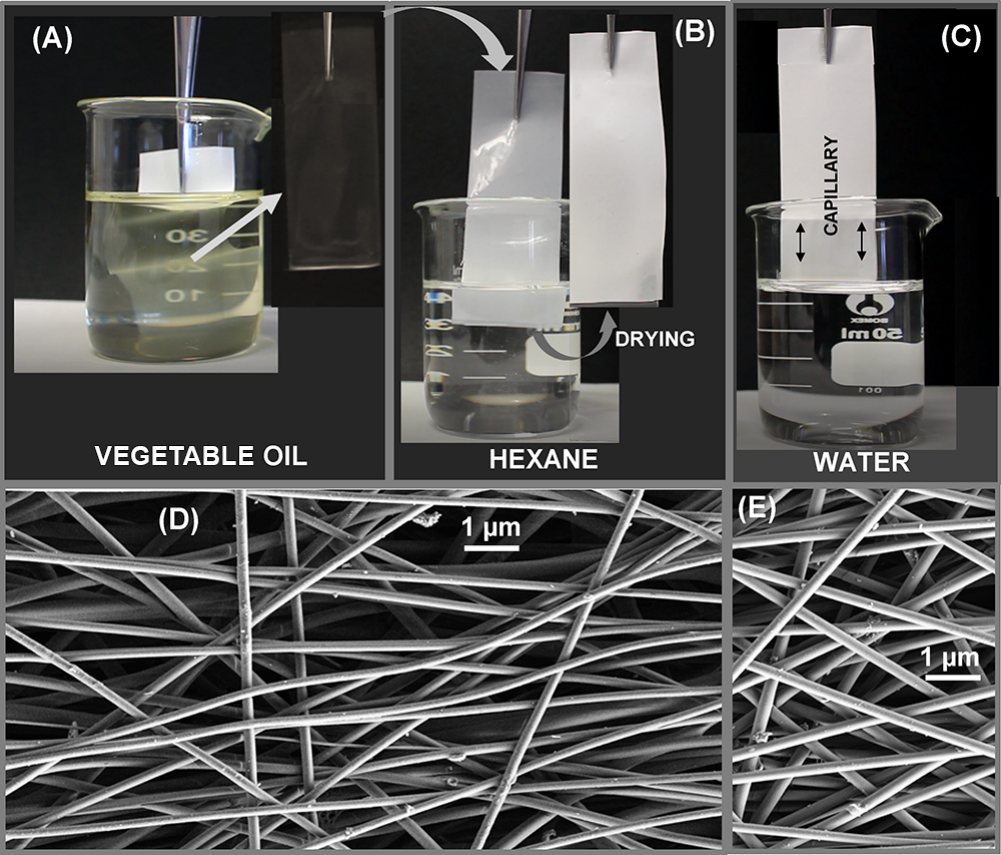
Membrane behavior in vegetable oil (sunflower oil) (A), oil removal by washing with hexane (B), and adsorption of water (*Q* = 2) immediately after oil removal (C). Membrane morphology before (D) and after (E) oil immersion and hexane washing.

After TT, the water adsorption capacity (*Q*) of the membrane was calculated by *Q* = absorbed mass of water/mass of dry membrane. To do so, six samples of different masses were dried in a vacuum oven at 50 °C for 2 h. Then, they were incubated in distilled water for 3 h at room temperature. Subsequently, the water retained on the surface of the samples was removed using a cellulose paper, the samples were weighed, and the value of *Q* was 2.06 ± 0.15. In addition, as shown in Figure 5d,e, TT does not cause any change in the morphology of the fibers. On the other hand, Figure 6 shows the irreversible amphiphilic character of the membrane after TT. Oil can penetrate and completely fill the interconnected pore structure of the membrane, displacing the air trapped in the pores. This produces isotropy in the optical properties (refraction index) of the membrane that becomes completely transparent (see Figure 6A), indicating that vegetable oil and polymeric fibers have practically the same refraction index. After the removal of oil with hexane, the membrane becomes opaque again (see Figure 6B) and maintains its hydrophilic properties (*Q* = 2; see Figure 6C). In summary, extreme changes in the polarity of the medium (oil ↔ water) do not modify the amphiphilic properties of the membrane or change its morphology (see Figure 6D,E).

The thermal resistance of the membrane was studied by immersing it in water at 100 °C for 24 h. The morphology of the fibers (see Figure S10) and the mass of the membrane (200 mg) were the same before and after heating at 100 °C for 24 h, indicating great robustness for applications where high temperatures are required.

In addition, to analyze the properties of the membranes for filtration processes, a vacuum filtration test using a series of aqueous suspensions of monodisperse hydrophilic particles with sizes ranging from 200 to 3000 nm in diameter was performed; monodisperse hydrophilic particles used for the test were synthesized according to our previous work.^76^ The membrane allowed the passage of particles with diameters of less than 2500 nm.

### 3.3 Characterization of Galactose Oxidase and Laccase Immobilized on Nanofiber Membranes

**Figure 7:**
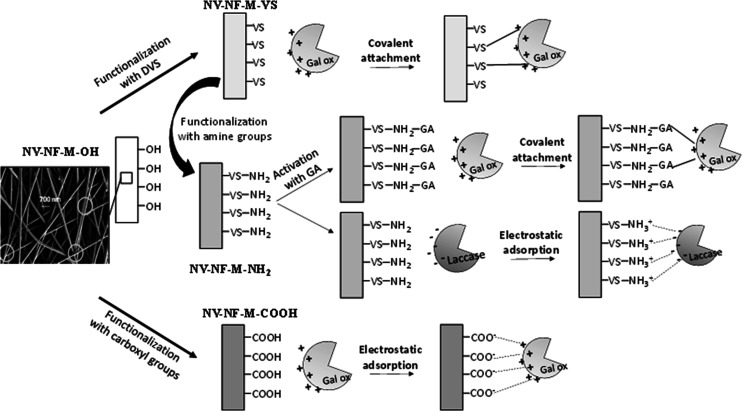
Different immobilization techniques used.

The usefulness of the nonwoven nanofiber membranes in enzyme immobilization was validated by studying different immobilization strategies for enzymes laccase and galactose oxidase (see Figure 7).

Evaluation of the immobilization followed standardized principles of enzyme catalysis.^12^ Results are shown in Table 2.

**Table 2:** Immobilization Parameters^a^ for the Immobilization of Laccase and Galactose Oxidase on Nonwoven Nanofiber Membranes with Different Surface Functionalization

surface functionalization	enzyme	immobilization strategy	surface activation	activity offered (U/g)	load (mg/g membrane)	yield (%)	activity observed (U/g)
DVS	gal ox	covalent attachment		353	6	66	46
carboxyl groups	gal ox	ionic adsorption	treated with PEI after immobilization				
			10%, average *M*_n_ ∼ 60,000	19	0.3	58	0.11
			1%, average *M*_n_ ∼ 60,000	35	0.5	52	0.36
			1%, average *M*_n_ ∼ 10,000	62	1.1	72	0.9
amino groups	gal ox	covalent attachment	preactivation with GA	21	0.3	66	0.3
				94	0.9	39	1.8
				1162	12.2	42	9.8
				7620	74.3	39	1.5
	laccase	ionic adsorption		21	10	85	12.5

a For more details, see Experimental S2.

Immobilization yield informs about the quantity of the immobilization based on the amount of enzyme quantified as activity balance (see the Supporting Information), while the measured activity informs about the functionality of the immobilized enzyme. Measurable activity quantifies the reaction rate of the heterogeneously catalyzed reaction by the membrane-immobilized enzyme (see Section 2 and the Supporting Information for more details). For covalent irreversible immobilization, a procedure of functionalization of NV-NF-Ms-OH with vinyl sulfone groups (RSOOCH_2_CH_2_) was established. After functionalization, the number of accessible vinyl sulfone groups was 281 ± 20 μmol/g; it was calculated by a luminescence assay with the fluorescent probe dansyl cadaverine. It is well known that vinyl sulfone groups can react easily with amine groups in mild conditions by a Michael-type reaction.^31,32,77^ Thus, the number of accessible vinyl sulfone groups of NV-NF-Ms-VS was calculated by a fluorescence assay. The assay includes the incubation of NV-NF-Ms-VS with the fluorescent probe dansyl cadaverine (DC) and subsequent quantification of DC in the supernatant via UV–Vis spectrometry. As a control, we use a membrane in which vinyl sulfone groups were previously blocked with ethanolamine. Controls were prepared by incubating NV-NF-Ms-VS in a 0.25 M solution of ethanolamine (NH_2_CH_2_CH_2_OH) in phosphate buffer at pH = 8 for 4 h. Then, samples and controls were incubated in a solution of 295 mg/L DC in phosphate buffer at pH = 8 for 4 h (see Figure S11). After incubation, the concentration of DC in the supernatant was quantified via UV–Vis spectrometry. The number of accessible vinyl sulfone groups in NV-NF-Ms-VS was calculated by subtracting the initial concentration of DC (*C*_0_ = 295 mg/L) and the concentration found in the supernatant (*C*_s_). Table 3 shows the results of the characterization. Table 3 also shows how the immobilization of DC in the controls was practically negligible, indicating an efficient blockade of the vinyl sulfone groups by ethanolamine.

**Table 3:** Characterization of the Number of Accessible Vinyl Sulfone Groups of NV-NF-Ms-VS

ref	supernatant absorbance (a.u.)	*C*_s_ (mg/L)	(*C*_0_ – *C*_s_) (mg/L)	membrane mass (mg)	mg DC/mg membrane	μmol DC/g membrane
sample-1	1.201	88.693	205.854	7.9	0.091	271.755
sample-2	1.274	94.021	200.525	7.5	0.094	278.839
sample-3	1.054	77.963	216.583	7.7	0.098	293.346
control-1	1.927	283.372	11.175	7.3	0.005	15.965
control-2	1.898	279.138	15.408	7.7	0.007	20.870
control-3	1.928	283.518	11.029	7.0	0.006	16.432

Figure S12 shows samples and controls under a UV lamp (350 nm) after incubation with DC at different times: 10, 20, 45, and 240 min. Once surface activation was achieved, immobilization of galactose oxidase was assessed following reported procedures.^49,50^ Results are shown in Table 2. A 66% immobilization yield of galactose oxidase (gal ox) was achieved (6 mg/g, 233 U/g) even when high loadings of enzyme were used. The recovered activity yielded 20%, which represents a significant value given the difficulties of active surface binding of gal ox and the scarce examples found in the literature.^12,78^ Further enhancement of the enzyme activity would require consideration of surface or microenvironment effects on the enzyme, especially the implementation and optimization of the surface blocking to erase the reactivity of remaining vinyl sulfone surface groups.^49^ Immobilization of laccase on vinyl sulfone-activated membranes did not give quantitative success; this is not surprising given the difficulty with the covalent attachment of laccase based on binding procedures that aim at nucleophilic surface residues, and the high glycosylation degree of laccase of *T. versicolor* could explain it.^79,80^

To study immobilization based on ionic adsorption driven by ion exchange, two strategies of surface functionalization were implemented. The membrane was functionalized with carboxyl and primary amine groups (see Sections 2.4.2 and 2.4.3). The concentration of accessible COOH groups was 560 ± 50 μmol/g; it was calculated by the toluidine blue O adsorption assay (TBO method).^81^ The TBO assay includes the incubation of carboxylate matrixes with toluidine blue O in alkaline buffer with subsequent washing, followed by elution and quantification of eluted TBO via UV–Vis spectrometry. Since reactivity between vinyl sulfone groups and amine groups is extremely efficient,^77,82,83^ the number of accessible amino groups was considered equal to the number of vinyl sulfone groups. The choice between carboxyl-activated (cation exchanger) and amine-activated (anion exchanger) membranes depends on the surface charges of the selected enzymes, which represent different exemplary cases. Whereas laccase displays a low p*I* (surface richer in anionic residues),^84^ galactose oxidase displays a high p*I* (surface richer in cationic residues).^85^ Therefore, different functionalization of the surface was necessary (see Figure 7). The activity offered, immobilization yield, and measured activity, known as immobilization parameters, are shown in Table 2. Different approaches are briefly discussed as follows.

Immobilization of galactose on the carboxyl-activated membrane was studied. Although the immobilization yield was higher than 50%, low binding stability was detected, and enzyme leaking was noticed (results not shown). To prevent this phenomenon, the immobilized biocatalyst was treated with different percentages and types of poly(ethyleneimine) (PEI) after the immobilization step. Results are shown in Table 2. Even though the immobilization yield is high, the final activity of the immobilized enzyme is far from the maximum. Tuning of the post-immobilization step (polymer coating) and/or fine surface features (density of functional groups), together with suitable regeneration of the enzyme through the catalytic cycles, are hypothesized as critical parameters.^86^ In the case of laccase, immobilization on amine-activated membranes was studied. As expected, immobilization proceeds with a high yield and great recovery of the catalytic activity (see Table 2). In that way, an easy and practical procedure of laccase adsorption leading to high recovered activity was found and was competitive with previous examples in other material formats.^57,59−63^

Finally, given the good results of the covalent attachment of galactose oxidase, another strategy of covalent immobilization of galactose oxidase was assessed. Table 2 shows that galactose oxidase was immobilized on membranes with functional amino groups, which were preactivated with glutaraldehyde. The enzyme was successfully bound into the membranes, allowing unprecedented high loads of the enzyme. The immobilization yield depends on the activity offered, decreasing with increasing amount of enzyme as expected.^12^ The effect of binding on the enzyme structure or surface effects due to the rich cationic character of the activated membranes could be responsible for the activity loss. Tuning of immobilization conditions (temperature and time) and fine surface features (density of functional groups) are hypothesized as critical parameters.

The enhancement of the results obtained and the further application of the membranes would be benefitted by the conjoint fine-tuning of the membrane structure and immobilization chemistry to achieve control on the enzyme structure and mass transfer phenomena through the mmebranes.^18,87−89^

## 4 Conclusions

In this work, we have exploited the mechanical properties of acrylate and methacrylate copolymers to manufacture (without additives or chemical crosslinking processes) an innovative generation of hydroxylated nonwoven nanofiber membranes NV-NF-Ms by electrospinning. To do so, ultrahigh-molecular-weight methacrylate and acrylate copolymers with the same chemical composition, (MMA)_1002_-*co*-(HEMA)_1002_ (*M*_w_ = 230,855 ± 7418 Da; *M*_n_ = 115,748 ± 35,567 Da; PDI = 2.00) and (MA)_11709_-*co*-(HA)_7806_ (*M*_w_ = 1.972 × 10^6^ ± 33,729 Da; *M*_n_ = 1.395 × 10^6^ ± 35,019 Da; PDI = 1.41), were designed, synthesized, and characterized by ^1^H-NMR and GPC. Then, we have shown that the polymeric blend (MMA)_1002_-*co*-(HEMA)_1002_/(MA)_11709_-*co*-(HA)_7806_ (50/50, w/w) is an excellent candidate to manufacture (without additives or chemical crosslinking processes) hydroxylated nonwoven nanofiber membranes (NV-NF-Ms-OH; 300 nm in fiber diameter) with enhanced mechanical and chemical properties such as high abrasion resistance, high flexibility and elasticity, high elongation at break, resistance to high temperatures, and easy manipulation: it can be cut, bent, twisted, etc.; this polymeric blend allows amphiphilic membranes with a high concentration of primary hydroxyl groups.

On the more fundamental side, we have also demonstrated that copper-mediated living radical polymerization (Cu^0^-MC-LRP) is also an excellent tool to design polar acrylate copolymers with ultrahigh molecular weight and perfectly adjusted polarity (concentration of hydroxyl groups). The primary hydroxyl groups present in the membranes can be easily activated with a battery of functional groups to provide different versatile enzyme immobilization chemistries from covalent immobilization to ionic adsorption based on ion exchange. In this way, vinyl sulfone-, amine-, and carboxyl-activated membranes were activated and suitability for immobilization was proven for two enzymes of biotechnological interest. Galactose oxidase and laccase immobilized on membranes were prepared with high enzyme loading and high recovered activity, demonstrating the practical application.

Last, we would like to remark that the membranes manufactured by electrospinning with the blend (MMA)_1002_-*co*-(HEMA)_1002_/(MA)_11709_-*co*-(HA)_7806_ (50/50, w/w) could be used for several applications such as water filtration and purification, oil/water separation, sensing and biosensing, and immobilization of metal catalysts, among others.

## Supporting Information Available

Electrospinning setup; theoretical modeling of copolymerization of monomers MA and HEA; chromatographic profile of acrylate copolymers; p(MA)-*co*-p(HEA); ^1^H-NMR spectra of acrylate copolymers; p(MA)-*co*-p(HEA); solubility of the copolymer (MA)_11709_-*co*-(HEA)_7806_ in different organic solvents; theoretical modeling of copolymerization of monomers MMA and HEMA; ^1^H-NMR spectra and chromatographic profile of the methacrylate copolymer; miscibility between copolymers (MA)_11709_-*co*-(HEA)_7806_ and (MMA)_1002_-*co*-(HEMA)_1002_ in DMF; elongation at break and abrasion resistance; morphology of the membrane before and after heating at 100 °C for 24 h; different immobilization techniques used; blocking of vinyl sulfone groups of NV-NF-Ms-VS; samples and controls under a UV lamp (340 nm) after incubation with DC at different times; concentration of each component in the copolymerization mixture; chemical composition of the copolymers; calculation of the immobilization parameters (PDF)
