# Supplementary material for: Hydrophilic Nonwoven Nanofiber Membranes as Nanostructured Supports for Enzyme Immobilization
Source: ACS Appl Polym Mater. 2022 Jul 22;4(8):6054–66. doi: 10.1021/acsapm.2c00863 (PMC9379912; doi:10.1021/acsapm.2c00863)
Supplement: Supplementary file 1 — ap2c00863_si_001.pdf [file ap2c00863_si_001.pdf]

Hydrophilic Non-woven Nanofibre Membranes as Nanostructured Supports for Enzymes Immobilization

Antonio L. Medina-Castillo, *[a,b] *Lucija Ruzic* [a,c,e], *Bernd Nidetzky* [d,e], Juan M. Bolivar *[c]

[a]*Nanomateriales y Polimeros S.L., (NanoMyP®), Spin-Off Company of the University of Granada, BIC Building, Avd. Innovacion 1, E-18016, Granada, Spain. Phone: +34 958637114, Fax: +34 958637114.*

[b]*Department of Analytical Chemistry, University of Granada, Avd. Fuentenueva s/n, 18071 Granada, Spain. E-mail:* antonioluismedina@ugr.es

[c]*FQPIMA group, Chemical and Materials Engineering Department, Faculty of Chemical Sciences, Complutense University of Madrid, Madrid, 28040, Spain. E-mail: juanmbol@ucm.es*

[d]*Austrian Centre of Industrial Biotechnology, Krenngasse 37, A-8010 Graz, Austria*

[e]*Institute of Biotechnology and Biochemical Engineering, Graz University of Technology, NAWI Graz, Petersgasse 12, A-8010 Graz, Austria*

index.

Fig.S1. Electrospinning set-up.

Fig.S2. Theoretical modelling of copolymerization of monomers MA and HEA.

Fig.S3. Chromatographic profile of acrylate copolymers; p(MA)-co-(HEA).

Fig.S4. ^{1}H-RMN spectra of acrylate copolymers; p(MA)-co-p(HEA).

Fig.S5. Solubility of copolymer $(MA)_{11709}$-co-$(HEA)_{7806}$ in different organic solvents.

Fig.S6. Theoretical modelling of copolymerization of monomers MMA and HEMA.

Fig.S7. ^{1}H-RMN spectra and chromatographic profile of methacrylate copolymer.

Fig.S8. Miscibility between copolymers $(MA)_{11709}$-co-$(HEA)_{7806}$ and $(MMA)_{1002}$-co-$(HEMA)_{1002}$ in DMF.

Fig-S9. Elongation at break and abrasion resistance.

Fig.S10. Morphology of the membrane before and after heating at 100ºC for 24 h.

Fig.S11. Blocking of vinyl sulfone groups of NV-NF-M-VS.

Fig. S12. Samples and controls under UV lamp (340 nm) after incubation with DC at different times.

Table S1. Concentration of each component in the copolymerization mixture.

Table S2. Chemical composition of the copolymers.

Experimental. S2. Calculation of the immobilization parameters.

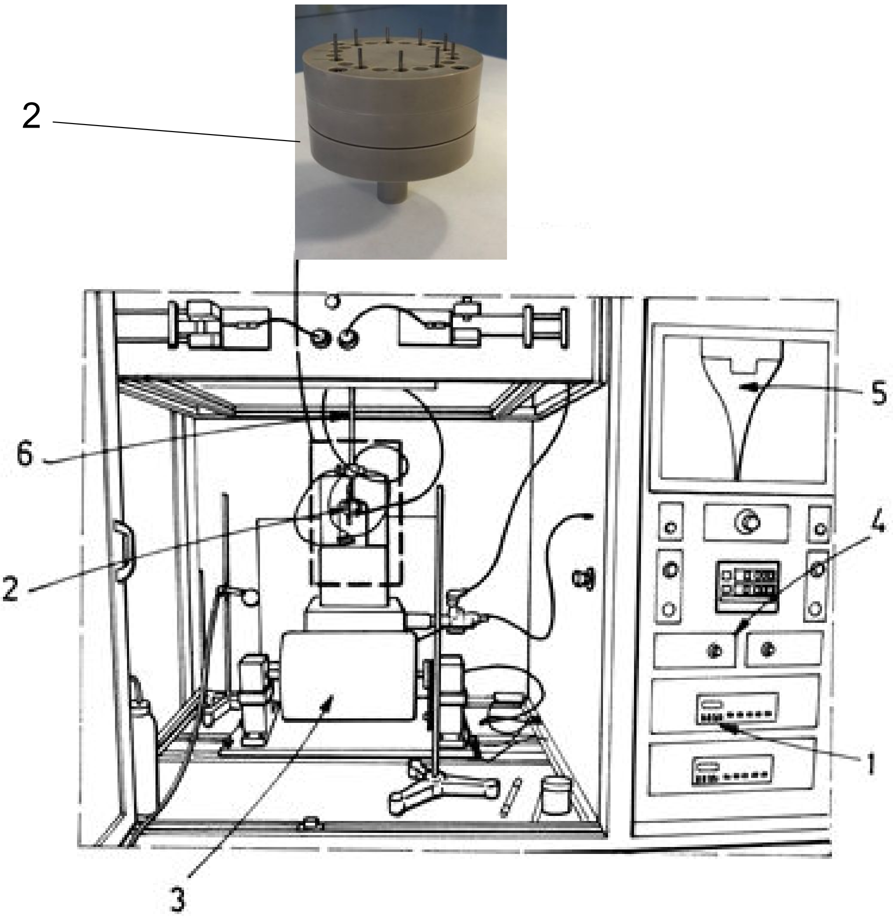

Fig. S1. Electrospinning set-up: injection pump (1), Ten-needle head (2), Drum collector (3), High voltage sources (4), Taylor cone display (5), and mechanical axis with axial movement (6).

Electrospinning parameters

Parameter	***Value***
Polymer concentration	*6 %*
Potential difference (ΔV)	*8 kV*
Flow rate (Q)	*5 ml/h*
Drum rotation speed (ω)	*500 rpm*
Distance between electrodes (δ)	*15 cm*
Temperature (T)	*20-25˚C*
Relative humidity (H.R.)	*25-30%*
Processing time (t)	*4h*
Axis movement (mm)	*±15 cm*

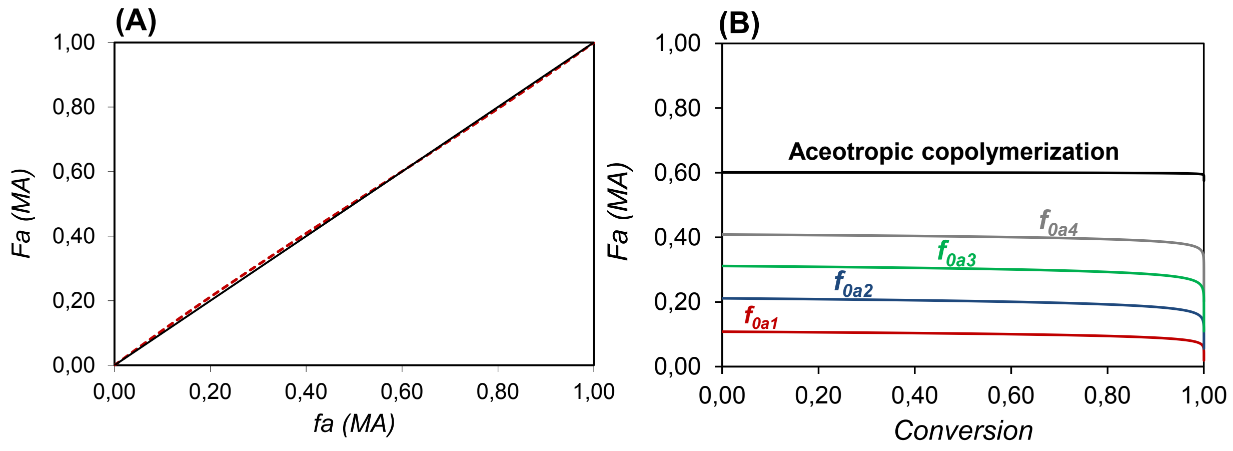

Fig. S2. Theoretical modelling of copolymerization of monomers MA and HEA, *Fa vs fa* (A) and *Fa vs Conversion* (B).

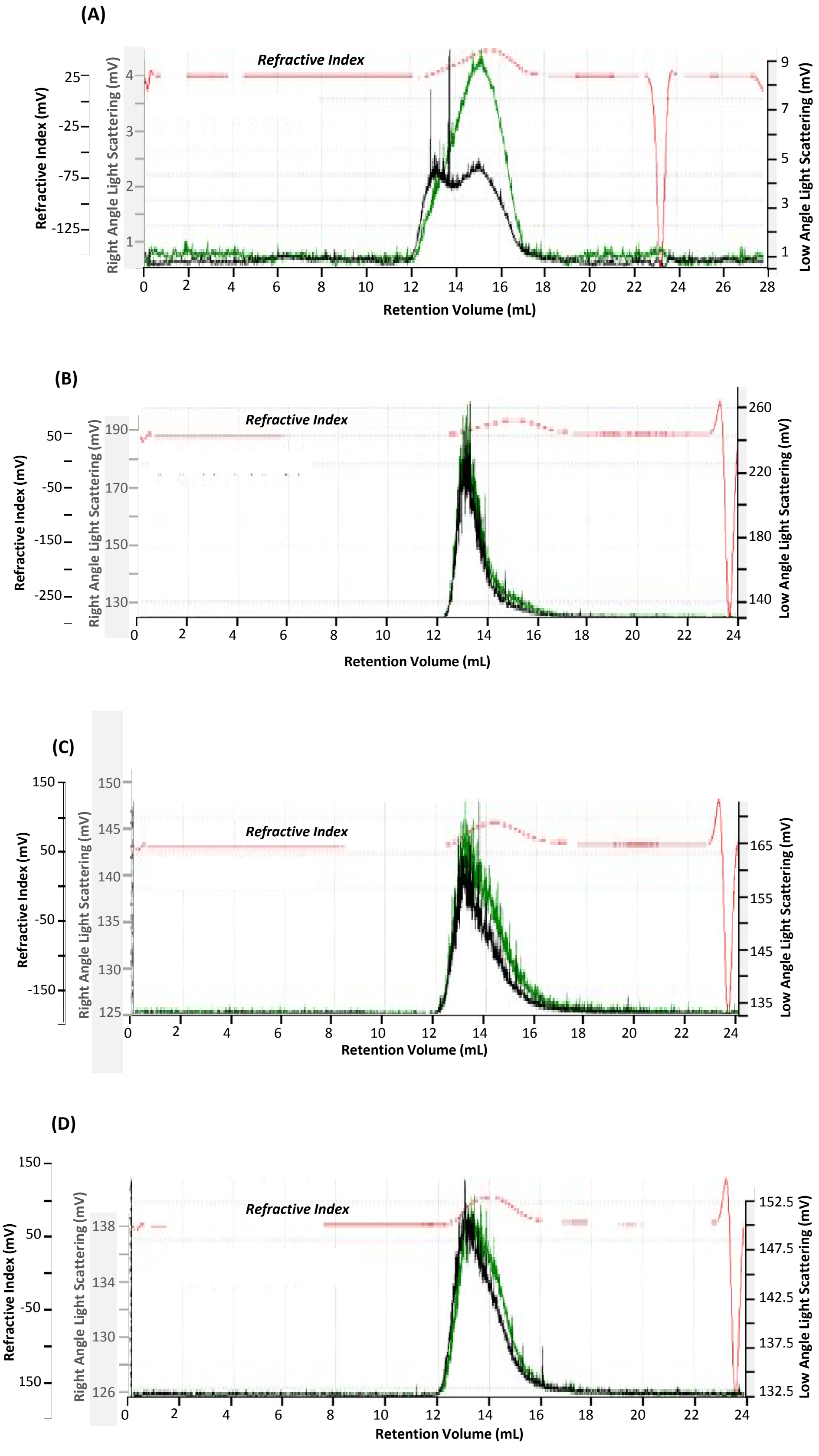

Fig.S3. Chromatographic profile of acrylate copolymers synthesised with different initial molar ratio %HEA/%MA; 10/90 (A), 15/85 (B), 25/75 (C), and 35/65 (D).

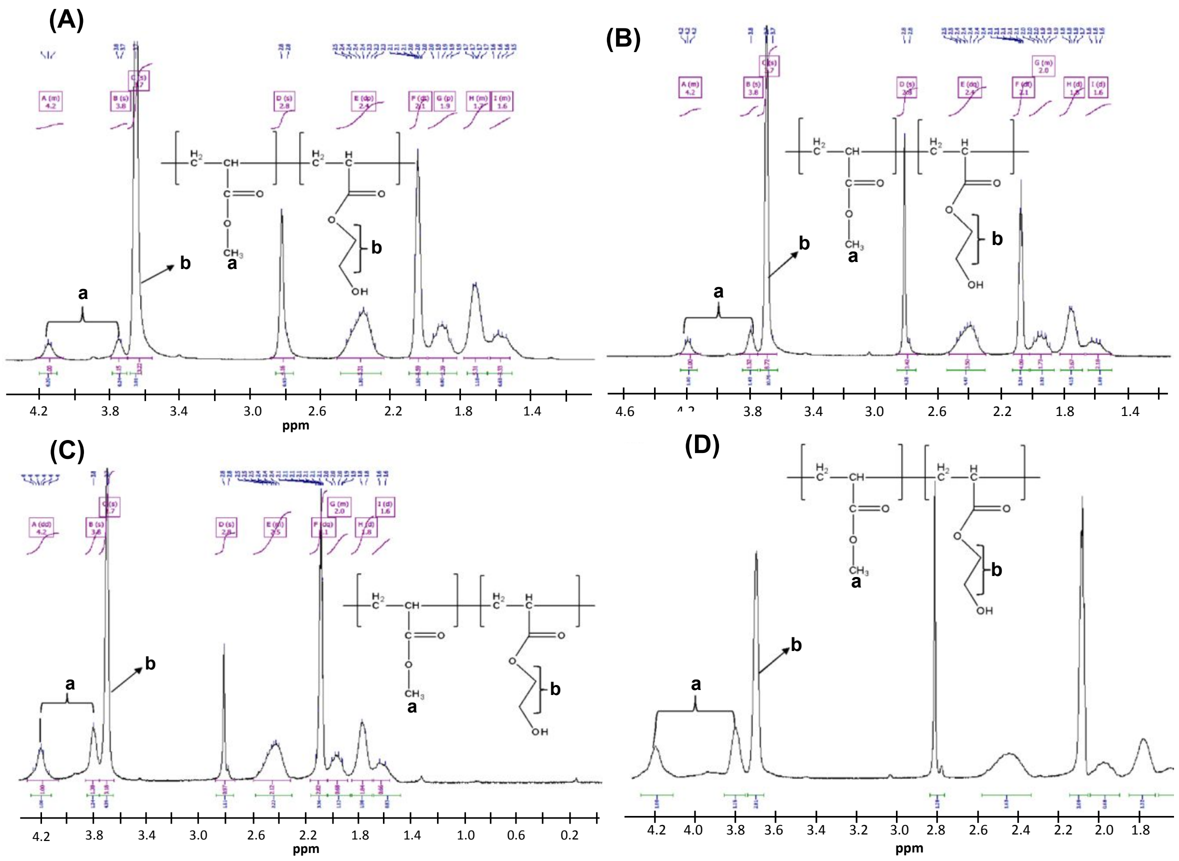

Fig.S4. ^{1}H-RMN spectra of copolymers p(MA)-co-p(HEA) synthesised with different initial molar ratio %HEA/%MA: 10/90 (A), 15/85 (B), 25/75 (C), and 35/75 (D).

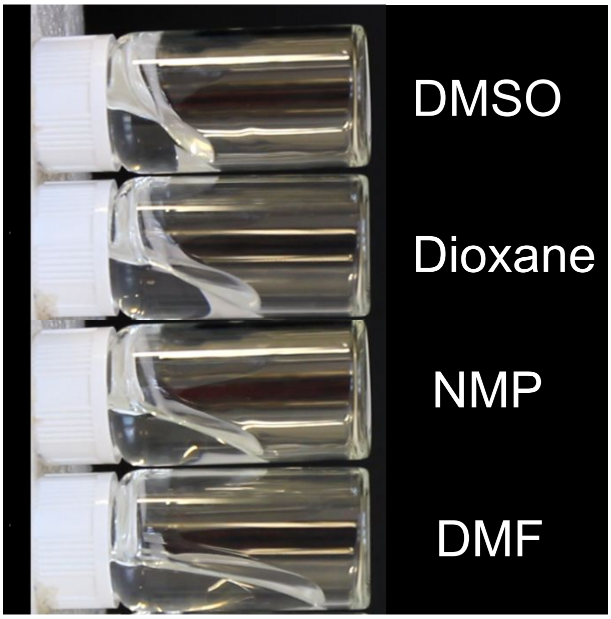

Fig.S5. Solubility of copolymer $(MA)_{11709}$-co-$(HEA)_{7806}$; 6% wt, in different organic solvents.

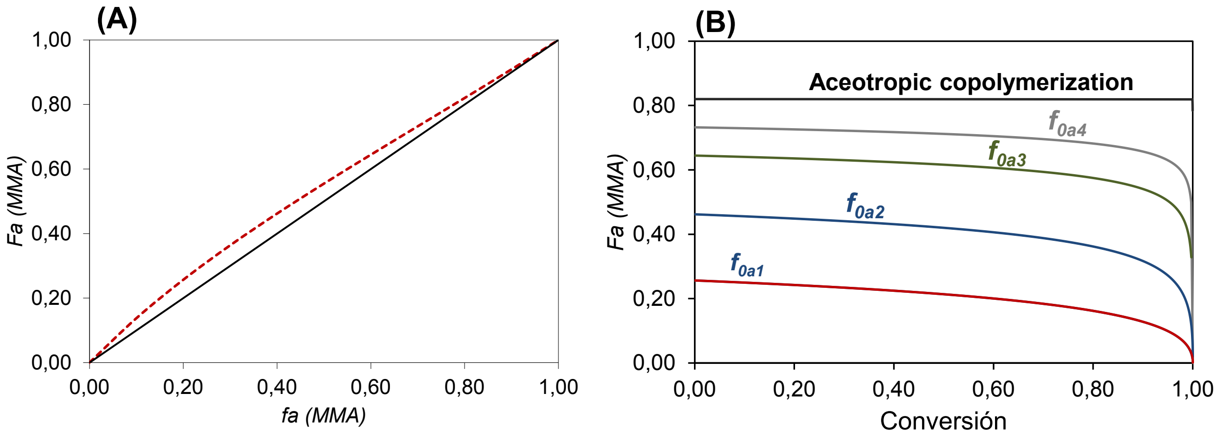

Fig.S6. Theoretical modelling of co-polymerization of monomers MMA and HEMA, *Fa* vs *fa* (A) and *Fa* vs *Conversion* (B).

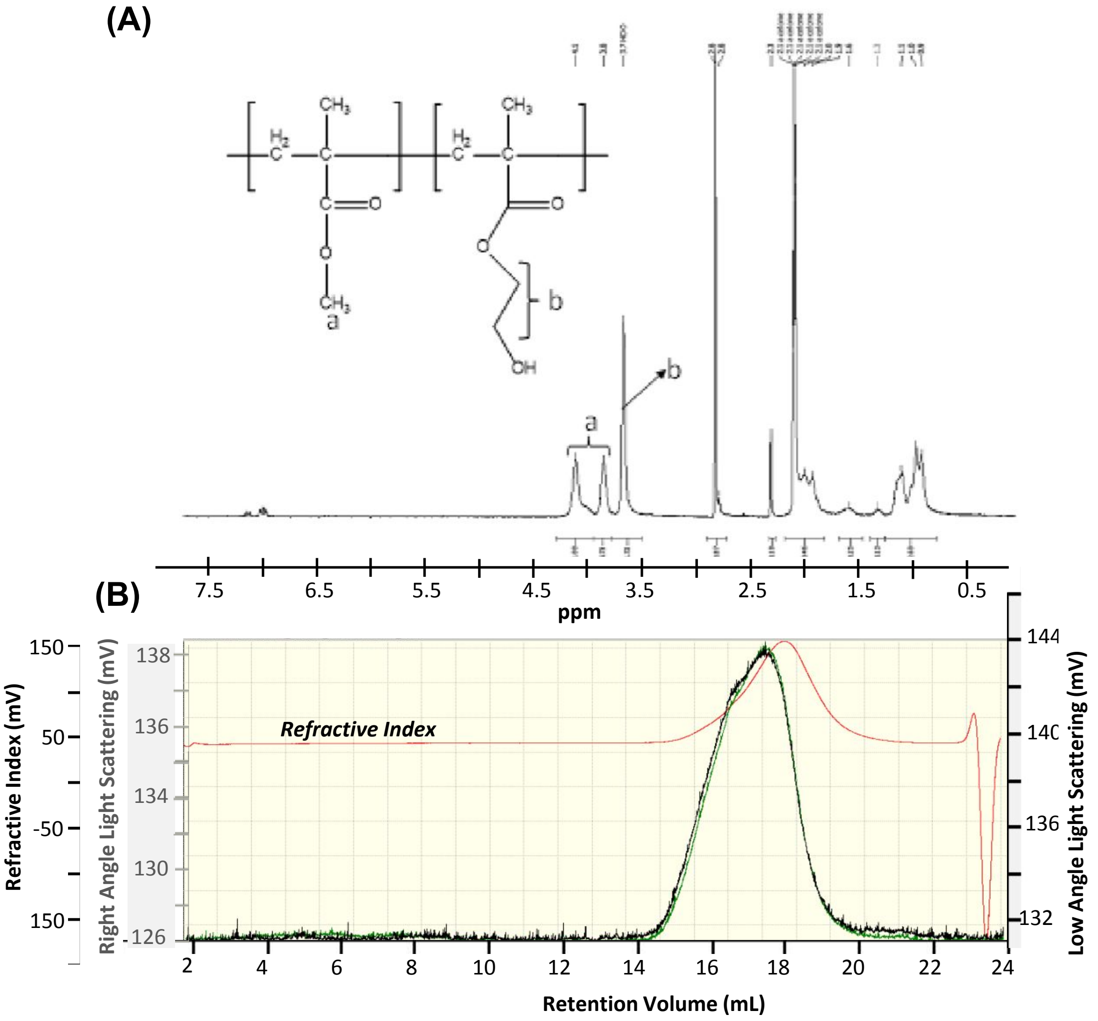

Fig.S7. ^{1}H-RMN spectra (A), and chromatographic profile (B) of copolymer (MMA)$_{1002}$-co-(HEMA)$_{1002}$.

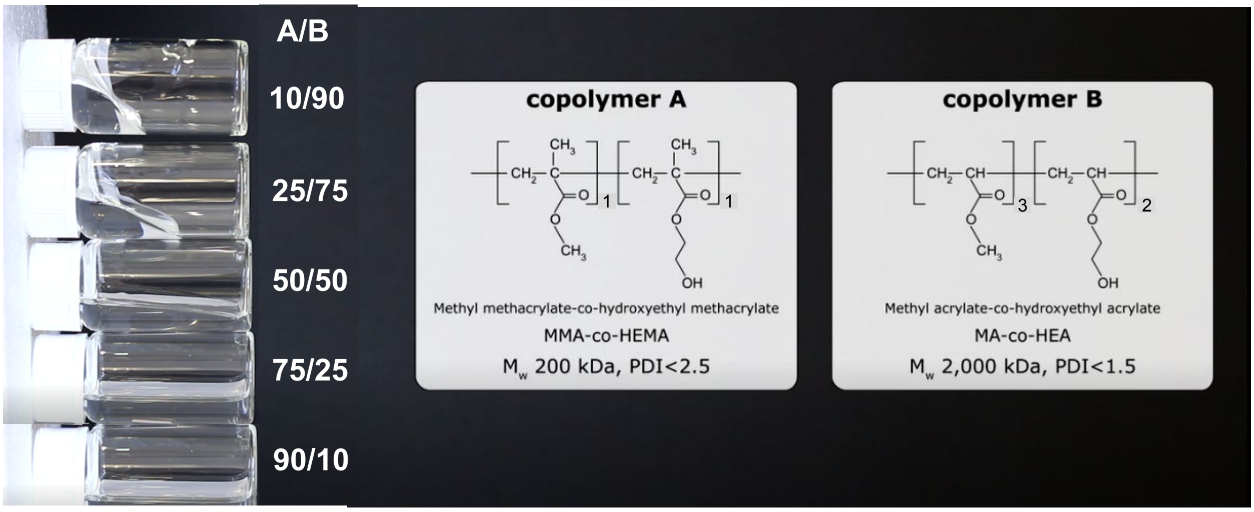

Fig.S8. Miscibility between copolymers $(MA)_{11709}$-co-$(HEA)_{7806}$ and $(MMA)_{1002}$-co-$(HEMA)_{1002}$ in DMF.

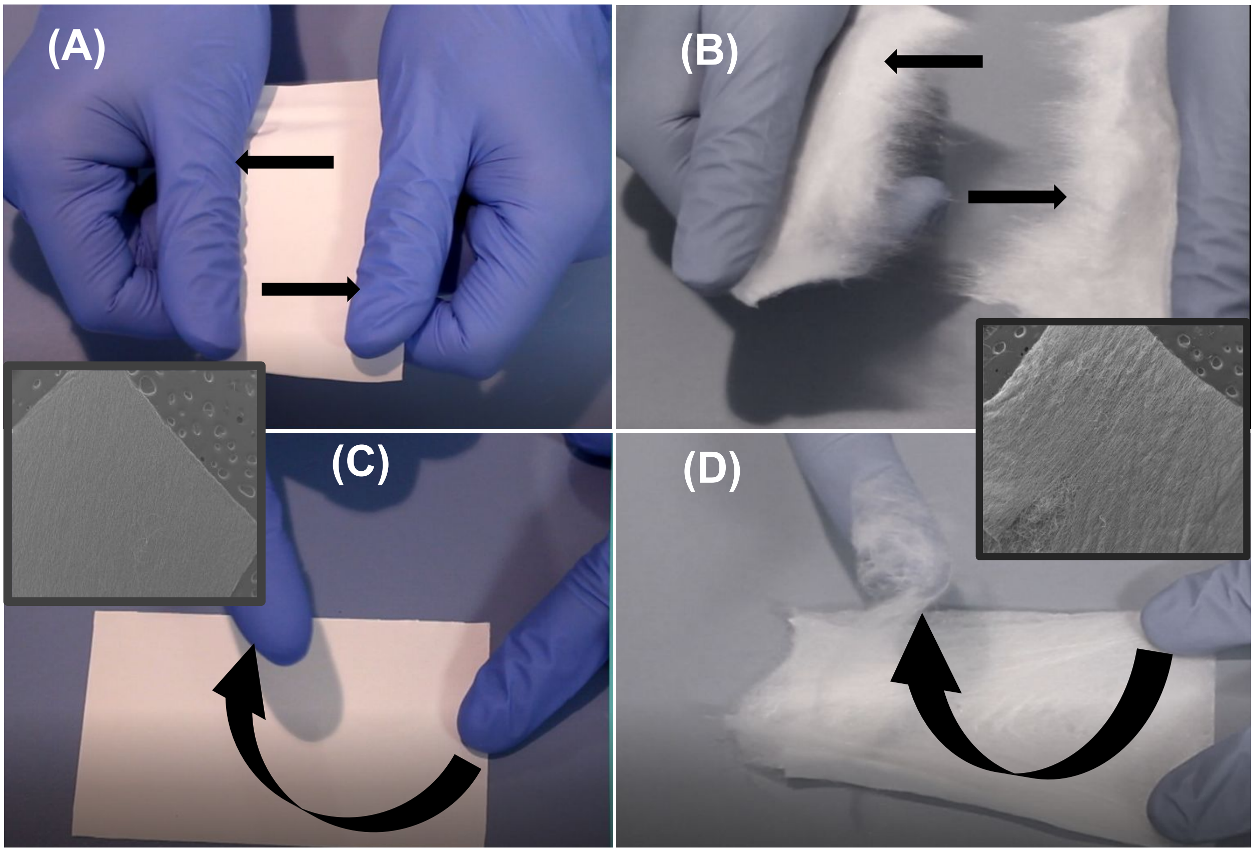

Fig.S9. Elongation at break and abrasion resistance of the membranes processing with blend $(MMA)_{1002}$-co-$(HEMA)_{1002}$/$(MA)_{11709}$-co-$(HA)_{7806}$ 75:25, w/w; (B**)** and (D), and with blend $(MMA)_{1002}$-co-$(HEMA)_{1002}$/$(MA)_{11709}$-co-$(HA)_{7806}$ 50:50, w/w; (A) and **(**C).

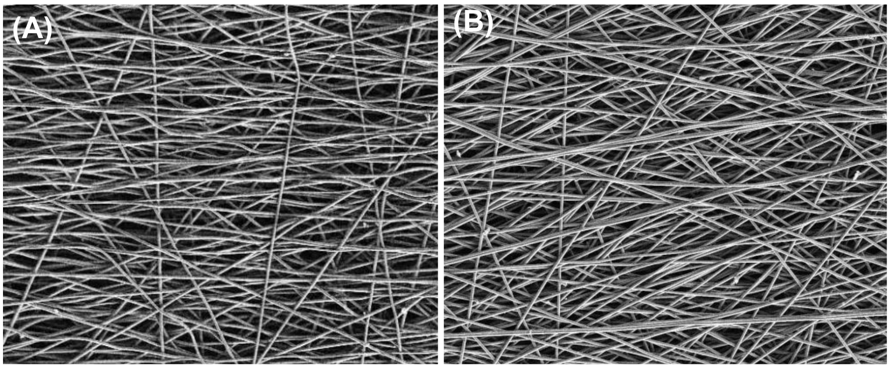

Fig.S10. Morphology of the membrane before and after heating at 100ºC for 24 h.

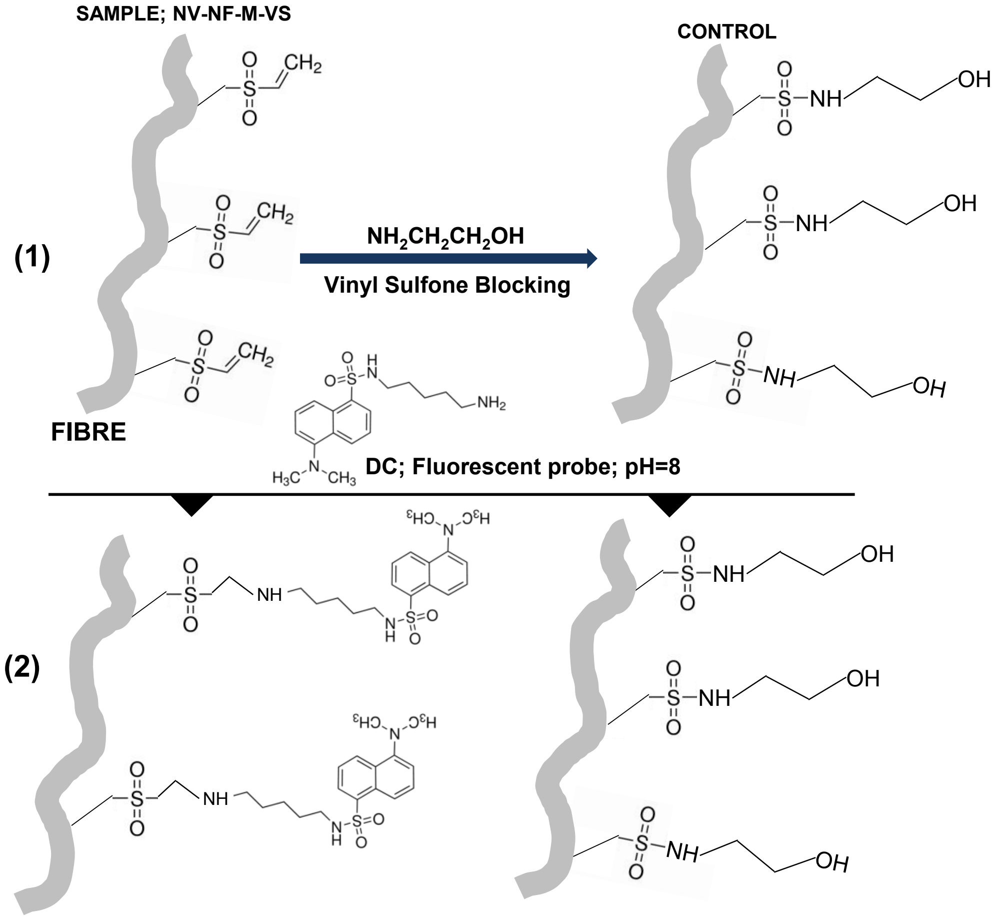

Fig. S11. Blocking of vinyl sulfone groups of NV-NF-M-VS; controls preparation (1), incubation of sample and control with the fluorescent probe Dansyl Cadaverine (2).

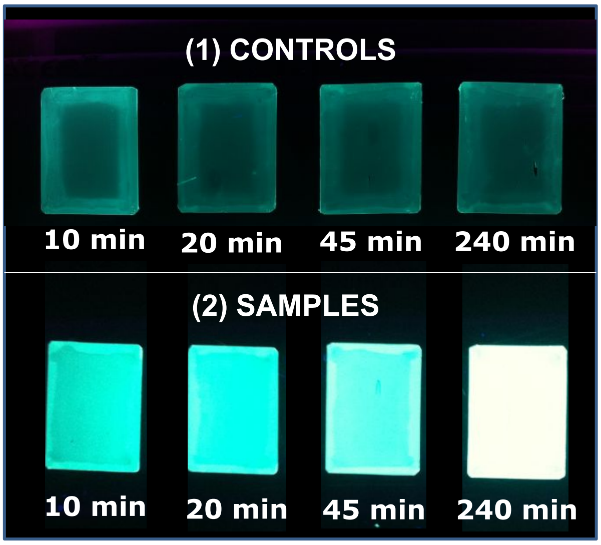

Fig. S12. Samples and controls under UV lamp (340 nm) after incubation with DC at different times.

Table S1. Concentration of each component in the copolymerization mixture.

Feed molar ratio (%HEA/%MA)	HEA %wt	MA %wt	DMSO %wt	MBP %wt	Cu^0 %wt	M6-TREN %wt	$CuBr_2$ %wt
10/90	6.20	43.78	49.99	0.0050	0.0017	0.0140	0.0010
15/85	9.70	40.27	49.99	0.0050	0.0017	0.0140	0.0010
25/75	15.56	34.42	49.99	0.0050	0.0017	0.0140	0.0010
34/66	20.37	29.62	49.99	0.0050	0.0017	0.0140	0.0010
45/55	23.88	26.11	49.99	0.0050	0.0017	0.0140	0.0010
55/45	28.82	21.17	49.99	0.0050	0.0017	0.0140	0.0010

Table S2. Chemical composition of the copolymers.

**Feed molar % HEA**	**Integral signal b**	**Integral signal a**	**Ratio MA:HEA in the copolymer**	**Molar % HEA in the copolymer**
10	0.44	3.01	9:1	10
15	2.43	10.76	6:1	14
25	2.24	4.39	5:2	28
35	2.15	2.81	3:2	36

Experimental. S2. Calculation of the immobilization parameters.

Immobilization yield (Y_I) and effectiveness factor (η) were calculated as shown in equations [1] and [2].

$$Y_I = \frac{(A_{offered} - A_{unbound})}{A_{offered}} \times 100\% \qquad [1]$$

$A_{offered}$ - enzyme activity in the enzyme solution that was offered to the carrier (U ml^{-1})
$A_{unbound}$ - enzyme activity in the supernatant (U ml^{-1})

$$\eta = \frac{A_{measured}}{A_{offered} \times \frac{Y_I}{100}} \times 100\% \qquad [2]$$

$A_{measured}$ - enzyme activity measured on carrier particles (U g^{-1})
$A_{offered}$ - enzyme activity that was offered to the carrier (U g^{-1})
Y_I - immobilization yield
